# Early initiation of SGLT2 inhibitors is important, irrespective of ejection fraction: SOLOIST‐WHF in perspective

**DOI:** 10.1002/ehf2.13148

**Published:** 2020-12-22

**Authors:** Subodh Verma, Stefan D. Anker, Javed Butler, Deepak L. Bhatt

**Affiliations:** ^1^ Division of Cardiac Surgery, St. Michael's Hospital University of Toronto 30 Bond Street Toronto ON M5B 1W8 Canada; ^2^ Department of Cardiology (CVK) and Berlin Institute of Health Centre for Regenerative Therapies (BCRT) Charité‐Universitätsmedizin Berlin, and the German Centre for Cardiovascular Research (DZHK) Partner Site Berlin Germany; ^3^ Department of Medicine University of Mississippi School of Medicine Jackson MI USA; ^4^ Brigham and Women's Hospital Heart and Vascular Center Harvard Medical School Boston MA USA

Worsening heart failure (WHF) requiring hospitalization represents a vulnerable period for patients with heart failure.^1^, ^2^ Such individuals, irrespective of the aetiology of heart failure, are at heightened risk of urgent heart failure visits, recurrent heart failure hospitalizations (HHF), and death. The early phase management of such patients is focused on the relief of congestion (with intravenous loop diuretics), stabilization of haemodynamics, and optimization of tissue perfusion. As clinical stabilization is achieved and successful weaning from intravenous to oral therapies has begun, this pre‐discharge phase is characterized by optimization of evidence‐based therapies, which in the case of heart failure with reduced ejection fraction (HFrEF) includes angiotensin‐converting enzyme inhibitors/angiotensin receptor blockers/angiotensin receptor neprilysin inhibitors, β‐blockers, and mineralocorticoid receptor antagonists.^3^ These disease‐modifying therapies, when instituted early, serve to markedly reduce morbidity and mortality following discharge.^4^, ^5^, ^6^, ^7^, ^8^, ^9^

Sodium‐glucose cotransporter 2 (SGLT2) inhibitors, originally described as therapies for hyperglycaemia, have now emerged as powerful tools to reduce heart failure outcomes in patients with HFrEF.^10^, ^11^, ^12^, ^13^, ^14^, ^15^, ^16^, ^17^, ^18^, ^19^ In two recently completed studies—DAPA‐HF (Study to Evaluate the Effect of Dapagliflozin on the Incidence of Worsening Heart Failure or Cardiovascular Death in Patients With Chronic Heart Failure) and EMPEROR‐Reduced (EMPagliflozin outcomE tRial in Patients With chrOnic heaRt Failure With Reduced Ejection Fraction)—dapagliflozin and empagliflozin, respectively, reduced the risk of cardiovascular death and HHF by 26% in patients with HFrEF.^20^, ^21^, ^22^ Importantly, these benefits were observed consistently in those with and without type 2 diabetes, were in addition to excellent background heart failure therapies, and resulted in an improvement in patient reported quality of life indices.^20^, ^21^, ^22^, ^23^, ^24^, ^25^, ^26^, ^27^, ^28^, ^29^, ^30^, ^31^, ^32^ Because both trials recruited patients with chronic ambulatory HFrEF [and excluded patients with hospitalization due to decompensated heart failure less than 4 weeks prior to enrolment (DAPA‐HF)], it can be argued that the question of in‐hospital initiation of SGLT2 inhibitors during a WHF event has remained unanswered. In addition, whether SGLT2 inhibitors would be beneficial irrespective of ejection fraction was unclear.

To address these two questions, the SOLOIST‐WHF (Effect of Sotagliflozin on Cardiovascular Events in Patients With Type 2 Diabetes Post Worsening Heart Failure) trial was conducted.^33^ A total of 1222 patients with type 2 diabetes [with an estimated glomerular filtration rate (eGFR) ≥ 30 mL/min/1.73 m^2^] who had been recently hospitalized for WHF were studied. Patients had to be treated with intravenous diuretics during the index hospitalization, and prior to randomization had to be stable, off intravenous inotropes, off oxygen, and had to have transitioned to oral diuretics. In addition, participants had to have either a brain natriuretic peptide level of ≥150 pg/mL (≥450 pg/mL if atrial fibrillation was present) or an N‐terminal pro‐brain natriuretic peptide level of ≥600 pg/mL (≥1800 pg/mL if atrial fibrillation was present). Eligible patients were randomized to receive the SGLT2/SGLT1 inhibitor sotagliflozin (200 mg once daily with up‐titration to 400 mg as tolerated) vs. placebo either before or within 3 days of discharge. Approximately 20% of patients randomized had an ejection fraction of ≥50%, and the median eGFR was ~50 mL/min/1.73 m^2^. Half of the patients received their first dosing while still in the hospital and the other half within 3 days following discharge. The ultimate primary endpoint of SOLOST‐WHF—which in order to preserve statistical power was changed because of the premature closure of the study due to loss of funding from the sponsor during the onset of the COVID‐19 pandemic—was the composite of total HHF, urgent heart failure visits, or cardiovascular deaths. It was reduced by 33% in those receiving sotagliflozin vs. placebo [hazard ratio (HR) 0.67; 95% confidence interval (CI) (0.52, 0.85), *P* = 0.0009]. This translated into a number needed to treat of four patients for a year. The cumulative incidence curves for the primary outcome separated early and were significant by day 28 post‐randomization. Efficacy was consistent across baseline ejection fraction (*Figure* *1*), eGFR categories (above and below 60 mL/min/1.73 m^2^), and in those who received initial therapy in‐hospital vs. within 3 days of being discharged. The time to first event of either cardiovascular death or HHF was reduced by 29% [HR 0.71; 95% CI (0.56, 0.89); *P* = 0.003]. The hazard ratio for cardiovascular death was 0.84 [(95% CI 0.58, 1.22); *P* = 0.36].

**Figure 1:**
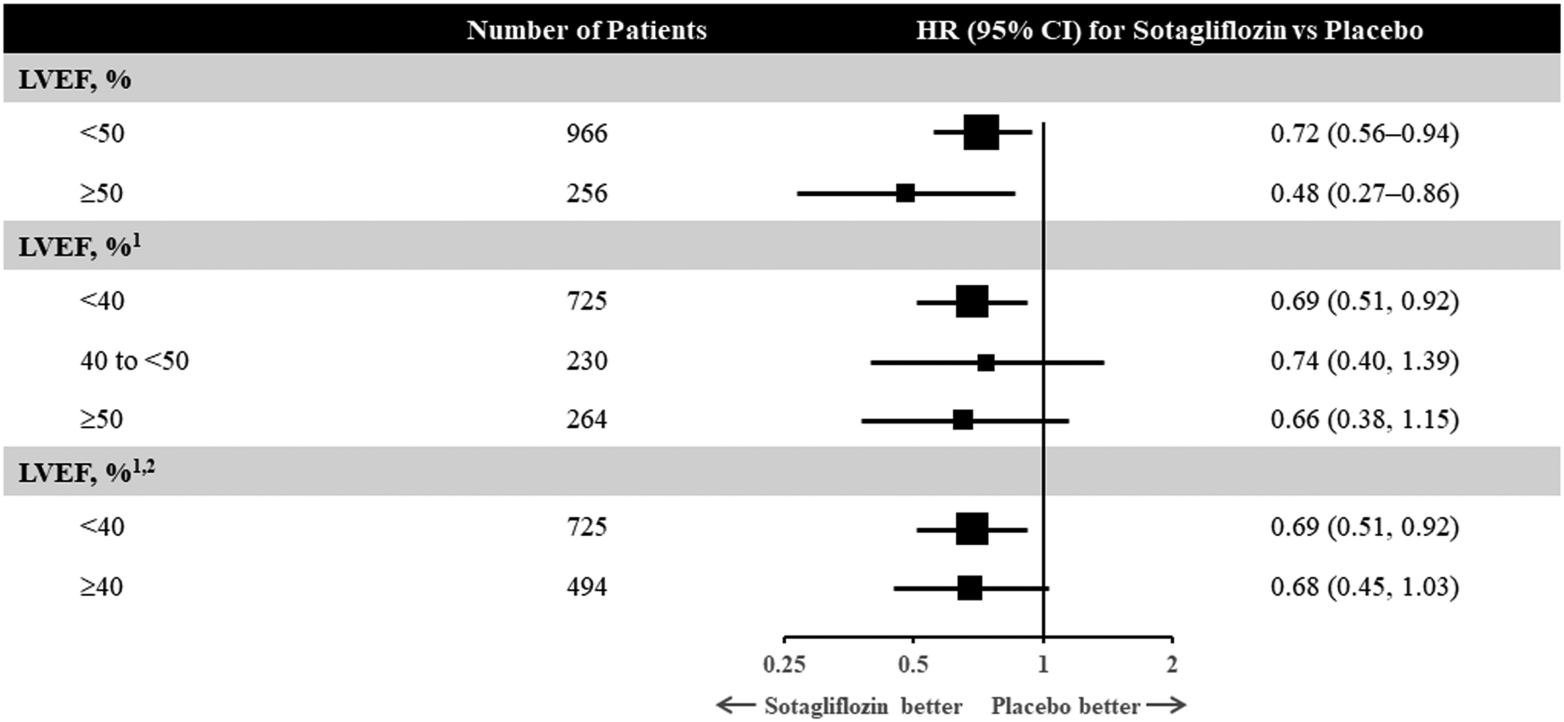
Primary efficacy outcome of SOLOIST‐WHF (composite of total hospitalization for heart failure, urgent heart failure visits, or cardiovascular deaths) as stratified by LVEF. LVEF, left ventricular ejection fraction.^1^Classification based on available baseline LVEF value and does not correspond exactly with categories defined by the randomization stratification factor, which is shown in the top two rows of the figure. Randomization in the study was stratified by baseline LVEF (<50% and ≥50%), and the actual baseline value was also recorded in the study case report form. After unblinding of the database, it was noted that there were a small number of inconsistencies between the classification of patients based on the randomization stratification factor and available baseline LVEF value. Specifically, 3 patients randomized in the <50% stratum did not have an available LVEF value in the database, 9 patients with available LVEF value <50% were randomized in the ≥50% stratum, and 17 patients with available LVEF value ≥50% were randomized in the <50% stratum. ^2^Post hoc. Adapted from Bhatt *et al*.^33^

The trial, which originally planned to enrol ~4000 patients, had to be truncated, as noted above. Despite this challenge, the primary outcome (both the original and the revised) demonstrated a meaningful and statistically significant benefit of sotagliflozin, although the trial had limited power to detect differences in mortality. Treatment was also associated with a significant improvement in quality of life as reported by a 4.1‐point increase in the Kansas City Cardiomyopathy Questionnaire‐12 score in sotagliflozin‐treated vs. placebo‐treated patients (*P* = 0.005). Beyond Week 4, the mean decrement in the eGFR was significantly lower in the sotagliflozin arm vs. placebo (*P* = 0.02). There were no major safety issues with sotagliflozin. Diarrhoea (6.9% vs. 4.1%) and severe hypoglycaemia (1.5% vs. 0.3%) were higher in the sotagliflozin‐treated vs. placebo‐treated patients.

There are several important take‐aways from SOLOIST‐WHF. First, in people with type 2 diabetes admitted with WHF requiring intravenous diuretics, SGLT2 inhibitors should be initiated as soon as the patient is clinically stable, preferably prior to discharge (or within days of discharge) during the phase of oral therapy optimization (*Figure* *2*). Prior studies of SGLT2 inhibitors, namely DAPA‐HF and EMPEROR‐Reduced, focused primarily on ambulatory patients with chronic HFrEF with and without diabetes. SOLOIST‐WHF provides the first clinical trial evidence of efficacy of earlier initiation of SGLT2 inhibitors. The results of SOLOIST‐WHF raise the possibility that this effect is present irrespective of baseline ejection fraction. By stratification factor at randomization, 256 patients had an EF classified as ≥50%, and the HR was 0.48 (95% CI 0.27, 0.86). Of the 1222 patients enrolled, 725 had an available baseline ejection fraction of <40% while 230 and 264 had ejection fractions of 40–49% and ≥50%, respectively. The HR for the primary outcome was apparently similarly reduced in all groups (0.69, 0.74, and 0.66, respectively). Because the subgroup of patients with ejection fraction above 50% was modest in size, further data of SGLT2 inhibitors in heart failure with preserved ejection fraction (HFpEF) are eagerly anticipated.

**Figure 2:**
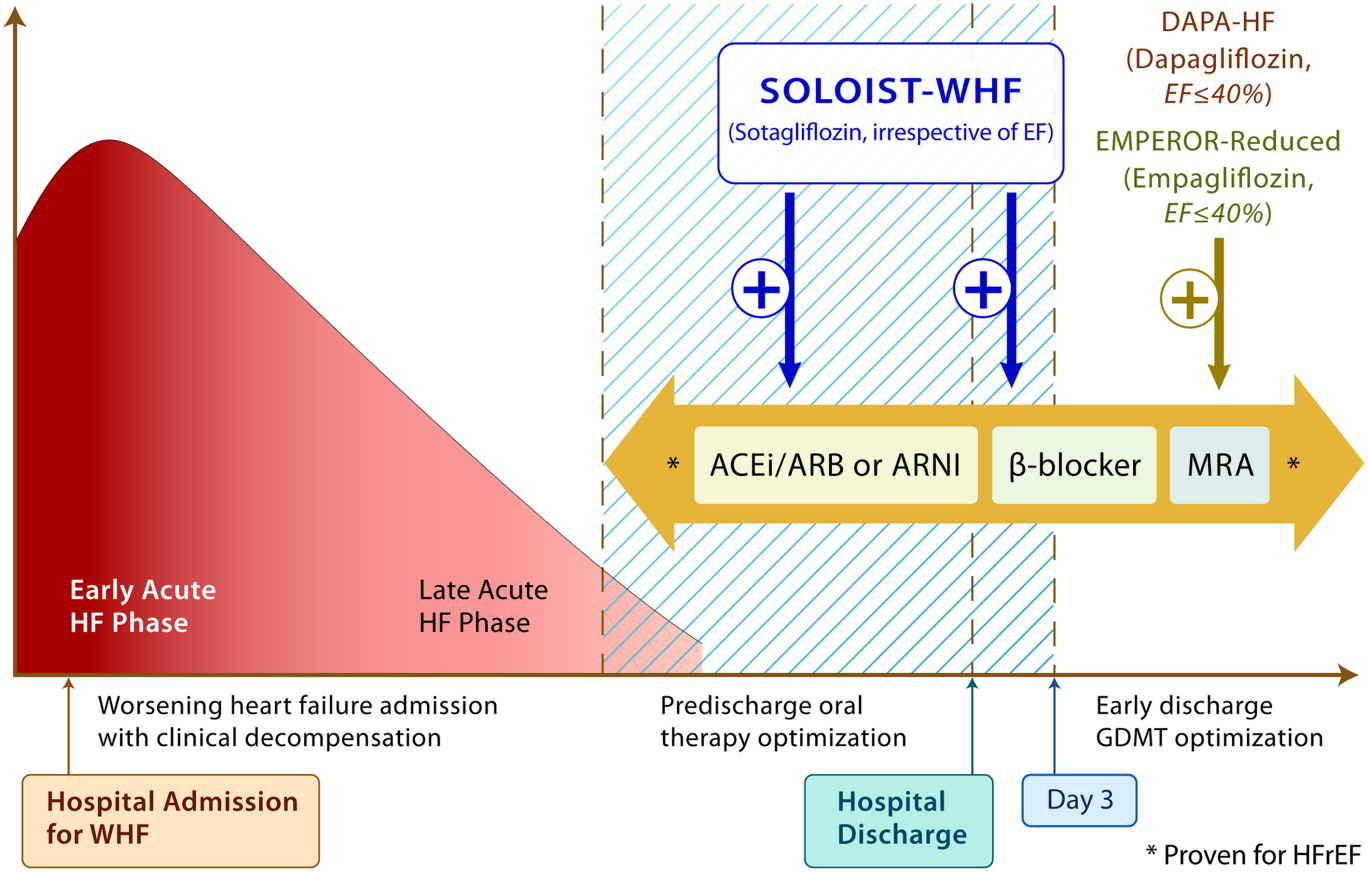
The natural history of worsening heart failure and the stages of worsening heart failure that have been evaluated in clinical trial settings. DAPA‐HF, Study to Evaluate the Effect of Dapagliflozin on the Incidence of Worsening Heart Failure or Cardiovascular Death in Patients With Chronic Heart Failure; EF, ejection fraction; EMPEROR‐Reduced, EMPagliflozin outcomE tRial in Patients With chrOnic heaRt Failure With Reduced Ejection Fraction; GDMT, guideline‐directed medical therapy; HFrEF, heart failure with a reduced ejection fraction; MRA, mineralocorticoid receptor antagonists; RASi, renin‐angiotensin system inhibitors; WHF, worsening heart failure.

The combined analysis of the SOLOIST‐WHF and SCORED (Effect of Sotagliflozin on Cardiovascular and Renal Events in Patients With Type 2 Diabetes and Moderate Renal Impairment Who Are at Cardiovascular Risk) trials further supports that there was a consistent benefit of sotagliflozin in HFpEF. The EMPEROR‐Preserved (EMPagliflozin outcomE tRial in Patients With chrOnic heaRt Failure With Preserved Ejection Fraction)^34^ and DELIVER (Dapagliflozin Evaluation to Improve the LIVEs of Patients With PReserved Ejection Fraction Heart Failure; ClinicalTrials.gov Identifier: NCT03619213) trials that are ongoing will provide valuable information in those with HFpEF in the ambulatory setting. Ongoing studies in patients with acute heart failure that include people with and without diabetes will help answer this question further (DAPA ACT HF‐TIMI 68, ClinicalTrials.gov Identifier: NCT04363697; EMPULSE, ClinicalTrials.gov Identifier: NCT04157751). While SOLOIST‐WHF studied patients with type 2 diabetes, evidence from both DAPA‐HF and EMPEROR‐Reduced point toward an entirely consistent benefit of dapagliflozin and empagliflozin in those with and without type 2 diabetes.^27^, ^31^ Therefore, it would be reasonable to hypothesize that the benefits noted in SOLOIST‐WHF would also extend to those without type 2 diabetes.

As expected, compared with DAPA‐HF and EMPEROR‐Reduced, patients enrolled in SOLOIST‐WHF were at higher risk. Indeed, the placebo event rate for first cardiovascular death or HHF (per 100 patient years) was 48% in SOLOIST‐WHF. In contrast, the placebo event rates in DAPA‐HF and EMPEROR‐Reduced were 15.3% and 21%, respectively, in the entire cohort and 25.5% and 28.5% in those with diabetes. These data further emphasize how diabetes in the context of heart failure is associated with worse clinical outcomes.^35^

Several mechanism(s) have been put forward to help explain the benefits of SGLT2 inhibition on heart failure.^14^, ^36^, ^37^, ^38^, ^39^ The early separation of the curves (within days)—which is also seen for dapagliflozin in DAPA‐HF^40^ and for empagliflozin in EMPEROR‐Reduced^30^ ^—^may point toward an important haemodynamic effect of SGLT2 inhibition in patients with heart failure. Indeed, recent human physiological studies in heart failure have indicated that SGLT2 inhibition promotes fractional sodium excretion within a few hours of treatment initiation—an effect that is exaggerated in the context of a loop diuretic.^41^ In acute heart failure, the EMPA‐RESPONSE‐AHF (Effects of Empagliflozin on Clinical Outcomes in Patients With Acute Decompensated Heart Failure) pilot study has shown an early effect on diuresis and reduction in whole body water content.^42^ Other relevant mechanisms may include an increase in erythropoietin,^43^ inhibition of the sympathetic nervous system,^36^ improved kidney function, changes in substrate utilization,^38^ direct myocardial effects, modulation of autophagy/mitophagy, and a stimulation of a fasting transcriptional paradigm.^44^ SGLT2 inhibitors have also been shown to have favourable effects on left ventricular remodelling in people with and without diabetes and in those with systolic or diastolic dysfunction.^45^, ^46^, ^47^, ^48^ While most of these mechanisms have been described with SGLT2 inhibitors per se, it is important to point out that sotagliflozin also inhibits SGLT1, which may have additional glycaemic and cardiovascular benefit.^49^ Indeed, in the SCORED study^50^ that was conducted in people with type 2 diabetes and chronic kidney disease, sotagliflozin was associated with significant haemoglobin A1C improvements at low eGFR levels (those at which conventional SGLT2 inhibitors do not demonstrate similar efficacy). SGLT1 inhibition may also produce distinct vascular effects, and that may explain the unique, early efficacy of sotagliflozin to reduce rates of ischaemic events (including stroke) in SCORED. The exact impact of the SGLT1 inhibition component of sotagliflozin in addition to its SGLT2 inhibitory effects may only be assessable in head‐to‐head studies.

In summary, SOLOIST‐WHF argues strongly for more upstream use of SGLT2 and SGLT2/1 inhibitors in patients with WHF. A hospitalization for heart failure marks a vulnerable phase wherein failure to initiate guideline‐directed medical therapy constitutes a major missed opportunity to reduce patient morbidity and mortality. In‐hospital initiation of such therapy is an independent predictor of better medication adherence and outcomes.^51^, ^52^ While recent data from the GWTG‐HF (Get With the Guidelines‐Heart Failure) registry suggest that a large proportion of patients with HFrEF would be candidates for SGLT2 inhibitor therapy,^51^, ^52^ the uptake of these life‐saving therapies remains poor. As we celebrate the successes of this class of agents,^53^ in addition to some of the emerging therapies for WHF (e.g. vericiguat, ferric carboxymaltose, and omecamtiv mecarbil),^54^, ^55^, ^56^ clinicians and patients are now faced with more choices than they ever had. How to integrate, optimize, and overcome inertia remains the Achilles heel of contemporary heart failure management.

## Conflict of interest

S.V. holds a Tier 1 Canada Research Chair in Cardiovascular Surgery and reports receiving research grants and/or speaking honoraria from Amarin, Amgen, AstraZeneca, Bayer, Boehringer Ingelheim, Bristol‐Myers Squibb, Eli Lilly, EOCI Pharmacomm Ltd, HLS Therapeutics, Janssen, Merck, Novartis, Novo Nordisk, Pfizer, PhaseBio, Sanofi, Sun Pharmaceuticals, and the Toronto Knowledge Translation Working Group. He is the President of the Canadian Medical and Surgical Knowledge Translation Research Group, a federally incorporated not‐for‐profit physician organization. S.D.A. reports receiving fees from Abbott, Bayer, Boehringer Ingelheim, Cardiac Dimension, Impulse Dynamics, Novartis, Occlutech, Servier, and Vifor Pharma, and grant support for IITs from Abbott and Vifor Pharma. J.B. is a consultant for Abbott, Amgen, Array, AstraZeneca, Bayer, Boehringer Ingelheim, CVRx, Eli Lilly, G3 Pharmaceutical, Impulse Dynamics, Innolife, Janssen, Luitpold, Medtronic, Merck, Novartis, Novo Nordisk, Relypsa, Sequana, StealthPeptide, and Vifor. D.L.B. serves as Chair of SOLOIST‐WHF and SCORED (with research funding paid to Brigham and Women's Hospital) and reports the following relationships—Advisory Board: Cardax, CellProthera, Cereno Scientific, Elsevier Practice Update Cardiology, Level Ex, Medscape Cardiology, MyoKardia, PhaseBio, PLx Pharma, Regado Biosciences; Board of Directors: Boston VA Research Institute, Society of Cardiovascular Patient Care, TobeSoft; Chair: American Heart Association Quality Oversight Committee; Data Monitoring Committees: Baim Institute for Clinical Research (formerly Harvard Clinical Research Institute, for the PORTICO trial, funded by St. Jude Medical, now Abbott), Cleveland Clinic (including for the ExCEED trial, funded by Edwards), Contego Medical (Chair, PERFORMANCE 2), Duke Clinical Research Institute, Mayo Clinic, Mount Sinai School of Medicine (for the ENVISAGE trial, funded by Daiichi Sankyo), Population Health Research Institute; Honoraria: American College of Cardiology (Senior Associate Editor, Clinical Trials and News, ACC.org; Vice‐Chair, ACC Accreditation Committee), Baim Institute for Clinical Research (formerly Harvard Clinical Research Institute; RE‐DUAL PCI clinical trial steering committee funded by Boehringer Ingelheim; AEGIS‐II executive committee funded by CSL Behring), Belvoir Publications (Editor in Chief, Harvard Heart Letter), Canadian Medical and Surgical Knowledge Translation Research Group (clinical trial steering committees), Duke Clinical Research Institute (clinical trial steering committees, including for the PRONOUNCE trial, funded by Ferring Pharmaceuticals), HMP Global (Editor in Chief, Journal of Invasive Cardiology), Journal of the American College of Cardiology (Guest Editor; Associate Editor), K2P (Co‐Chair, interdisciplinary curriculum), Level Ex, Medtelligence/ReachMD (CME steering committees), MJH Life Sciences, Population Health Research Institute (for the COMPASS operations committee, publications committee, steering committee, and USA national co‐leader, funded by Bayer), Slack Publications (Chief Medical Editor, Cardiology Today's Intervention), Society of Cardiovascular Patient Care (Secretary/Treasurer), WebMD (CME steering committees); Other: Clinical Cardiology (Deputy Editor), NCDR‐ACTION Registry Steering Committee (Chair), VA CART Research and Publications Committee (Chair); Research Funding: Abbott, Afimmune, Amarin, Amgen, AstraZeneca, Bayer, Boehringer Ingelheim, Bristol‐Myers Squibb, Cardax, Chiesi, CSL Behring, Eisai, Ethicon, Ferring Pharmaceuticals, Forest Laboratories, Fractyl, Idorsia, Ironwood, Ischemix, Lexicon, Lilly, Medtronic, MyoKardia, Pfizer, PhaseBio, PLx Pharma, Regeneron, Roche, Sanofi, Synaptic, The Medicines Company; Royalties: Elsevier (Editor, Cardiovascular Intervention: A Companion to Braunwald's Heart Disease); Site Co‐Investigator: Biotronik, Boston Scientific, CSI, St. Jude Medical (now Abbott), Svelte; Trustee: American College of Cardiology; Unfunded Research: FlowCo, Merck, Novo Nordisk, Takeda.
